# Caveolin 1 and Caveolin 2 are associated with breast cancer basal-like and triple-negative immunophenotype

**DOI:** 10.1038/sj.bjc.6604463

**Published:** 2008-07-08

**Authors:** S E Elsheikh, A R Green, E A Rakha, R M Samaka, A A Ammar, D Powe, J S Reis-Filho, I O Ellis

**Affiliations:** 1 Department of Histopathology, School of Molecular Medical Sciences, Nottingham, UK; 2 Department of Histopathology, Faculty of Medicine, Menoufiya University, Egypt; 3 Department of General Surgery, Faculty of Medicine, Cairo University, Egypt; 4 The Breakthrough Breast Cancer Research Centre, Institute of Cancer Research, London, UK

**Keywords:** caveolin 1, caveolin 2, immunohistochemistry, breast, basal-like

## Abstract

Caveolin-1 (CAV1) and caveolin 2 (CAV2) are the principal structural proteins of caveolae, sphingolipid and cholesterol-rich invaginations of the plasma membrane involved in vesicular trafficking and signal transduction. Over the recent years there has been controversy about their role in breast cancer and their suitability as markers of basal-like phenotype. Caveolin-1 and CAV2 protein expression was assessed on a tissue microarray containing 880 unselected invasive breast cancer cases, by means of immunohistochemistry. Caveolin-1 and CAV2 expression was observed in 13.4 and 5.9% of all breast cancer, respectively. Their expression was strongly associated with high histological grade, lack of steroid hormone receptor positivity (ER and PR), and expression of basal markers (basal cytokeratins, P63, P-cadherin). Furthermore, there was a significant association between CAV1 and CAV2 expression and basal-like phenotype. On univariate analysis only CAV2 had a prognostic impact on breast cancer-specific survival; however, this was not independent from other traditional markers on multivariate analysis. Our results demonstrate that both CAV1 and CAV2 are associated with basal-like phenotype. Further studies are warranted to determine whether they play an oncogenic role in basal-like/triple-negative breast cancer development or are just surrogate markers for this subgroup.

Caveolae are special invaginated microdomains of the plasma membrane found in the majority of mammalian cells and serve as membrane organising centres. Three members of the caveolin family (CAV1, CAV2, and CAV3) have been identified and they play a pivotal role in intracellular trafficking of cellular components and in signal transduction (Hnasko and Lisanti, 2003). Despite several studies on caveolins in cancer, especially CAV1, their role in cancer development and progression is still contentious.

Caveolin-1 and *CAV2* genes map to chromosome locus 7q31.1 near the D7S522 genetic marker, which is a known fragile site (FRA7G) (Engelman *et al*, 1998; Hayashi *et al*, 2001; Lee *et al*, 2002). Caveolin-1 was first thought to have tumour suppressor properties (Razani *et al*, 2001a, 2001b; Hnasko and Lisanti, 2003), based on the finding of an arguable inactivating point mutation in *CAV1*, the silencing of *CAV1* gene expression by promoter hypermethylation in breast cell lines and prostate tumour samples (Engelman *et al*, 1999; Hurlstone *et al*, 1999; Cui *et al*, 2001) and the apparent downregulation of *CAV1* in breast cancer (Chen *et al*, 2004; Park *et al*, 2005). In recent years, there has been increasingly more coherent data to suggest that CAV1 and CAV2 may also have oncogenic properties in breast (Hurlstone *et al*, 1999; Pinilla *et al*, 2006; Van den Eynden *et al*, 2006; Savage *et al*, 2007, 2008), prostate (Yang *et al*, 1998; Thompson *et al*, 1999), bladder (Rajjayabun *et al*, 2001; Fong *et al*, 2003), oesophageal (Kato *et al*, 2002; Ando *et al*, 2007), thyroid, pancreatic, non-small cell and squamous lung cancer (Kato *et al*, 2004; Sunaga *et al*, 2004).

Despite the controversy about the distribution of CAV1 and CAV2 in normal and invasive breast cancer (Yang *et al*, 1998; Hurlstone *et al*, 1999; Chen *et al*, 2004; Sagara *et al*, 2004; Park *et al*, 2005; Van den Eynden *et al*, 2006), recent studies confirmed the preferential expression of both genes and their proteins in normal myoepithelial cells (Pinilla *et al*, 2006; Savage *et al*, 2007, 2008). Furthermore Savage *et al*, have recently reported high prevalence of CAV1 and CAV2 expression in basal-like breast carcinomas (Savage *et al*, 2007, 2008), and observed *CAV1* gene amplification in a small subgroup of basal-like breast cancers (Savage *et al*, 2007, 2008). Moreover, these findings support Pinilla *et al* who described an association between CAV1 expression and sporadic basal-like breast cancers and familial BRCA1 tumours (Pinilla *et al*, 2006).

Our aims in this study were (1) to assess CAV1 and CAV2 prevalence in a well-characterised series of 880 cases of invasive breast carcinomas using high-throughput tissue microarray (TMA) technology and immunohistochemistry; (2) to determine whether CAV1 and CAV2 could be used as diagnostic markers to identify basal-like subtypes of invasive breast cancers, and (3) to assess if CAV1 and CAV2 have prognostic significance on the outcome of patients with invasive breast cancer.

## Materials and Methods

### Patient data

The tissue microarrays comprised a cohort of 880 consecutive breast tumours from patients diagnosed between 1986–1998 and entered into the Nottingham Tenovus Primary Breast Carcinoma Series. Histological tumour types comprised 449 invasive ductal carcinomas of no special type (NST), 182 tubular mixed carcinomas, 25 medullary carcinomas, 83 lobular carcinomas, 28 tubular carcinomas, eight mucinous carcinomas, six cribriform carcinomas, four papillary carcinomas, 29 mixed NST and lobular carcinomas, 23 mixed NST and special type carcinomas, and six miscellaneous tumours. Full details of the characterisation of the TMA and the cohort of the patients are described elsewhere (Elbauomy Elsheikh *et al*, 2007). Patient management was based on tumour characteristics provided by the Nottingham Prognostic Index (NPI) and hormone receptor status. Patients with an NPI score ⩽3.4 received no adjuvant therapy, those with a NPI score >3.4 received tamoxifen if oestrogen receptor (ER) positive: (±Zoladex if pre-menopausal) or classical cyclophosphamide, methotrexate and 5-fluorouracil if ER negative and fit enough to tolerate chemotherapy (Madjd *et al*, 2005). Tumours were graded according to a modified Bloom–Richardson scoring system (Elston and Ellis, 1991) and size was categorised according to the TNM staging criteria (Singletary and Connolly, 2006). Nottingham Prognostic Index was calculated as previously described (Galea *et al*, 1992). Survival data including disease-free survival (DFS), metastasis-free survival (MFS) and breast cancer specific survival (BCSS) were maintained on a prospective basis. Disease-free survival and MFS were defined as the interval (in months) from the date of the primary surgical treatment to the first loco-regional or distant recurrence, respectively. Breast cancer-specific survival survival was taken as the time (in months) from the date of the primary surgical treatment to the time of death from breast cancer. This study was approved by the Nottingham Research Ethics Committee 2 under the title of ‘Development of a molecular genetic classification of breast cancer’.

### Immunohistochemical staining and scoring

Breast cancer tissue microarrays were prepared and immunohistochemically (IHC) stained for CAV1 and 2 as described previously (Abd El-Rehim *et al*, 2004b, 2005a; Rakha *et al*, 2006; Reis-Filho *et al*, 2006). Validation of both antibodies was performed in previous studies (Savage *et al*, 2007, 2008). The mouse monoclonal antibodies (2297, ref. 10 at 1 : 150 dilution and clone 65 at a dilution of 1 : 50, both BD Transduction Labs, Erembodegem, Belgium) were used for CAV1 and CAV2 staining, respectively, following microwave heat-induced antigen retrieval using DAKO antigen retrieval solution (pH 6.0) (DakoCytomation, Glostrup, Denmark). Detection was achieved with the Envision kit (Dako). Negative controls comprising omission of the primary antibody and IgG-matched serum, were included in each IHC run. Caveolin-1 and CAV2 immunohistochemical distribution on tissue microarray sections was analysed by three of the authors (ER, RS & AA), separately. Cases were classified as positive if any membranous staining (with or without cytopalsmic reactivity) was found. The analysis was performed blinded to the results of other immunohistochemical markers and patients’ outcome. The immunostaining, morphometric scoring using H-score, and dichotomous categorisation of oestrogen receptor, progesterone receptor, cytokeratin (Ck) 7/8, Ck 18, Ck 19, Ck 5/6, Ck 14, HER2, EGFR, P63, E-cadherin and P-cadherin are described elsewhere (Abd El-Rehim *et al*, 2004a, 2004b, 2005b). On the basis of the expression of HER2, ER, Ck 5/6 and EGFR, tumours were classified according to the immunohistochemical panel proposed by Nielsen *et al* (2004) and to triple-negative phenotype (TN), which was immunohistochemically defined by the lack of expression of oestrogen receptor (ER), progesterone receptor (PR) and HER2 (HER2) (Bauer *et al*, 2007; Carey *et al*, 2007; Rakha *et al*, 2007).

### Statistical analysis

Statistical analysis was performed using SPSS 13.0 statistical software. Median follow-up was defined as follow-up period for those patients still alive and disease-free at their latest hospital visit. All factors were used as dichotomous covariates in the statistical analysis with the exception of grade, NPI and phenotypic groups proposed by Nielsen *et al* (2004) that were divided into three groups. Unweighted κ agreement coefficient test was used to assess agreement between observers of the same variables. To test whether these variables differed according to clinicopathological variables and biological markers the χ^2^ test and Fisher's exact test were used. All *P*-values were two-sided, and *P*<0.05 was considered significant. Kaplan–Meier plots were used to visualise the survival distribution. Differences in DFS, MFS and BCSS on the basis of CAV1 and CAV2 expression were estimated using log-rank test. Cox proportional hazards model was used to test the statistical independence and significance of predictors on DFS, MFS and BCSS.

## Results

### Patient clinical outcome

Follow-up data were available for 547 out off 561 cases that showed informative data for CAV1 or CAV2. Survival time ranged from 1 to 192 months (median – 86 months, mean – 81 months). During this period, a total of 78 (13.9%) patients died from breast cancer. Of the available cases, 106 (18.9%) cases were grade 1, 167 (29.8%) cases were grade 2, and 274 (48.8%) were grade 3. At the time of the primary diagnosis, 184 (32.8%) of the patients had lymph node-positive disease, 375 (66.8%) had tumour size more than 2 cm and distant metastases was observed in 87cases (15.5%).

### The incidence of CAV1 and CAV2 expression in invasive breast cancer

**Figure 1:**
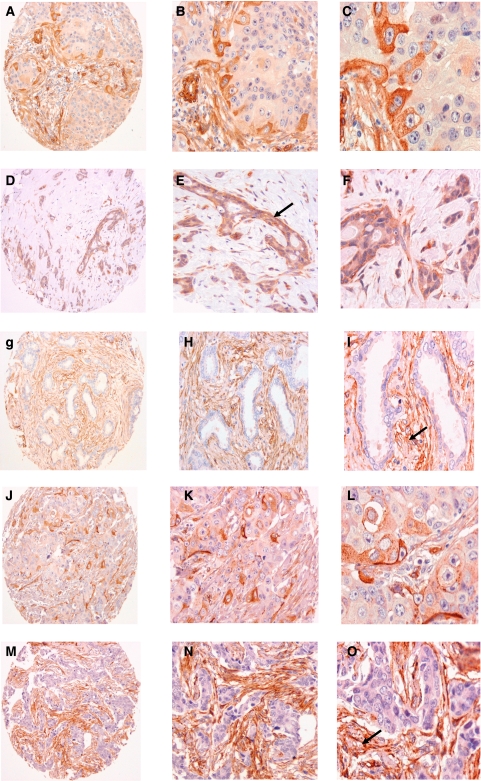
Caveolin-1 and CAV2 expression in invasive duct carcinoma (IDC) of the breast. (**A**–**C**) CAV1 expression in Grade 3 IDC. (**D**–**F**) CAV1 positive expression in Grade1 IDC the arrow in (**E**) point to the associated DCIS component. (**G**–**I**) CAV1 negative expression in Grade 1 IDC. (**J**–**L**) CAV2 positive expression in Grade3 IDC. (**M**–**O**) CAV2 negative expression in Grade 1 IDC. The arrows in (**I** and **O**) point to the positive stromal cells, which represent an internal positive control for CAV1 and CAV2. (**A**, **D**, **G**, **J** and **M** original magnification × 100, (**B**, **E**, **H**, **K** and **N**) original magnification × 200; (**C**, **F**, **I**, **L** and **O**) original magnification × 400).

After excluding the uninformative TMA cores, which were either lost, fragmented or did not have invasive tumour, 516 cases were analysable for CAV1 and/or CAV2: CAV1, *n*=461; CAV2, *n*=410; and both, *n*=310. Caveolin-1 positivity was detected in 13.4% whereas CAV2 was found in 5.9% of invasive breast cancer cases (Table 1) (Figure 1). Coexpression of both CAV1 and CAV2 proteins was found in only 2.5% out of 310 cases where informative data were available for both CAV1 and CAV2. A statistically significant correlation between the expression of both proteins was found (*P*<0.001). Good agreement was found between observers regarding CAV1 and CAV2 scoring (unweighted κ score=0.57879) (0.4678–0.6896).

### Correlation between CAV1 and clinicopathological variables and immunohistochemical markers

There was a significant positive correlation between CAV1 expression and high histological grade (*P*=0.026), lack of ER and PR expression (*P*<0.001 and 0.004, respectively). A significant inverse correlation between expression of luminal cytokeratins (Ck 7/8, Ck 18, and Ck 19) and positivity for CAV1 was found (*P*=0.003, 0.001, and 0.026, respectively). Furthermore, there was a strong positive association between CAV1 and basal cytokeratin (Ck5/6 and Ck14) (*P*=0.004 and 0.038, respectively) expression, as well as positivity for P63 and P-cadherin (*P*<0.001 and 0.007, respectively). These results were reflected in the phenotypic groups of breast cancer proposed by Nielsen *et al* (2004), where 40.5% of CAV1 positive cases had a basal-like phenotype, whereas 41.1% displayed a triple-positive phenotype (both *P*<0.001, Table 2).

### Correlation between CAV2 and clinicopathological variables and immunohistochemical markers

CAV2 expression was associated with poor prognostic parameters. CAV2 expression was associated with high tumour grade (*P*=0.005), large tumour size (*P*<0.001) and poor Nottingham prognostic index (*P*=0.017). A strong inverse correlation was found between lack of steroid hormone receptors ER, PgR, and AR with positive expression of CAV2 (*P*<0.001, <0.001 and 0.039, respectively). CAV2 positive breast cancers frequently expressed basal markers, including Ck 5/6, Ck 14, p63 (*P*<0.001, 0.001 and <0.001, respectively). CAV2 was more frequently negative in those that lacked expression of luminal cytokeratins, including Ck 7/8 and Ck 18 (*P*⩽0.001 and 0.022, respectively). An inverse correlation between CAV2 and E-cadherin expression was found (*P*=0.034). As expected, CAV2 displayed a strong association with basal-like immunophenotype as defined by Nielsen *et al*.'s criteria (Nielsen *et al*, 2004), where 66.7% of CAV2-positive cases were of basal type whereas 58.1% displayed a triple-negative phenotype (both *P*<0.001, Table 2).

### CAV1 and 2 expressions in relation to patient outcome

**Figure 2:**
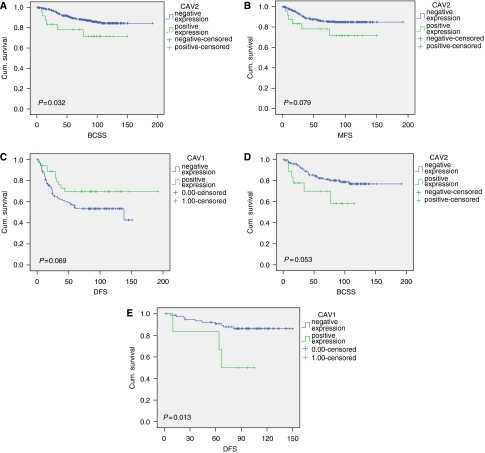
Kaplan–Meier plots for CAV1 and CAV2 expression in invasive breast cancer. (**A**, **B**) Whole cohort regarding BCSS and MFS for CAV2. (**C**, **D**) ER-negative cohort regarding DFS and BCSS with CAV1 and CAV2, respectively. (**E**) Low-grade cohort, the relation between CAV1 and DFS.

Kaplan–Meier survival analysis did not reveal any association between CAV1 expression and BCSS or DFS in the whole cohort. However, there was a significant association between positive CAV2 expression and shorter BCSS (*P*=0.032) and a trend for shorter metastasis-free survival (MFS, *P*=0.07). Further subgrouping of the cohort revealed that in the ER-negative group of patients CAV1 expression displayed a trend for longer DFS (*P*=0.069), whereas CAV2-positive cases showed a trend for shorter BCSS (*P*=0.053). The only significant relation between CAV1 expression and poor outcome was observed in the low tumour grade cohort of patients (Grade 1), where positive expression of CAV1 was associated with shorter DFS (*P*=0.013) (Figure 2). Although these results are of interest, it should be noted that this is a retrospective and exploratory analysis and the number of patients with grade 1, CAV1-positive breast cancers was rather limited. No statistically significant correlation was found between expression of CAV1, CAV2 and CAV1 and/or CAV2 and patient outcome (BCSS and DFS) in the group of chemotherapy-treated patients and when only patients with triple-negative breast cancers were analysed. On multivariate analysis, both proteins were shown not to be independent prognostic factors for DFS and BCSS.

## Discussion

Gene expression profiling has led to classification of breast cancers into five groups: luminal A, luminal B, basal-like, HER2+ and normal breast-like (Perou *et al*, 2000; Sorlie *et al*, 2001, 2003; van de Rijn *et al*, 2002; Sotiriou *et al*, 2003; Nielsen *et al*, 2004) and importantly these groups have prognostic and predictive implications. Basal-like tumours, which comprise approximately 15–20% of breast cancers, were so named because their transcriptome closely resembles that of myoepithelial/basal cells of normal breast. The majority of basal-like tumours lack ER, PgR and HER2 expression (i.e. display a triple-negative phenotype) (Nielsen *et al*, 2004; Rouzier *et al*, 2005; Banerjee *et al*, 2006; Livasy *et al*, 2006; Tan *et al*, 2007).

Previous studies using immunohistochemistry/immunofluorescence (Pinilla *et al*, 2006; Savage *et al*, 2007, 2008) and cDNA arrays (Jones *et al*, 2004; Sagara *et al*, 2004) have demonstrated that in ducts and lobules of normal breast, CAV1 and CAV2 expression is preferentially seen in myoepithelial and basal cells. However, caveolins 1 and 2 are also abundantly expressed in fibroblasts, adipocytes and endothelial cells of normal breast and breast cancers (Savage *et al*, 2007, 2008).

Given their distribution in normal breast, we assessed the expression of CAV1 and CAV2 in a large population of invasive breast cancer to determine whether they would be preferentially expressed in the subgroup of breast carcinomas with myoepithelial/basal-like phenotype and their prognostic implications in breast cancer.

This study highlights the link between CAV1 and CAV2 with the myoepithelial/basal- and triple-negative groups of breast cancer. The evidence for this comes from the correlation seen between CAV1 and CAV2 and other conventional basal markers (Ck 14, Ck 5/6, and P-cadherin). Furthermore, positive CAV1 and CAV2 expression was associated with poor prognostic parameters indicated by a poor Nottingham prognostic factor and high histological grade. Conversely, cases with positive hormone receptor status (ER and PR) and expressing luminal cytokeratins (Ck 7/8, Ck 18 and Ck 19) showed negligible expression of CAV1 and CAV2, which is in agreement with previous studies (Hurlstone *et al*, 1999; Charafe-Jauffret *et al*, 2006; Pinilla *et al*, 2006; Savage *et al*, 2007, 2008). Therefore, it is not altogether surprising to find a significant correlation between expression of caveolins 1 and 2 and basal-like phenotype defined by Nielsen *et al* (2004) (40.5 and 66.7% of CAV1- and CAV2-positive cases, respectively) and the TN phenotype (41.1 and 59.1% for CAV1- and CAV2-positive cases, respectively).

Taken together, these data suggest that expression of CAV1 and CAV2 is associated with basal-like phenotype in breast cancer. This is in accordance with previous *in situ* studies, which identified *CAV1* and *CAV2* genes as discriminators of the basal phenotype, and address them as one of the underlying mechanism driving CAV1 and CAV2 proteins overexpression in that group (Perou *et al*, 2000; Charafe-Jauffret *et al*, 2006; Savage *et al*, 2007, 2008). However, in previous gene expression profiling studies (Perou *et al*, 2000; Sorlie *et al*, 2001), *CAV1* and *CAV2* were shown to be expressed at higher levels in normal breast-like cancer. Interestingly, unlike CAV1, the level of CAV2 mRNA was also increased in the majority of basal-like cancer (Perou *et al*, 2000). Moreover, a PCR study suggested downregulation of CAV1 and CAV2 mRNA levels in non-microdissected breast cancer (Sagara *et al*, 2004). It should be noted, however, that analysis of the expression of caveolins 1 and 2 by expression array- and PCR-based methods should be interpreted with caution, given that the high levels of CAV1 and CAV2 reported in normal breast-like samples (Perou *et al*, 2000; Sorlie *et al*, 2001) may derive from stromal endothelial cells, fibroblast and adipocytes (Savage *et al*, 2007, 2008). Therefore, an accurate measurement of CAV1 and CAV2 mRNA levels in neoplastic cells is not possible without precise microdissection or by using *in situ* methods.

In this study, CAV1 expression was significantly associated with a shorter BCSS in patients with low grade invasive breast cancers whereas patients with CAV2-positive cancers had a shorter DFS. These findings are supported by previous reports showing that caveolin 1 and 2 expression is associated with highly aggressive tumours such as inflammatory breast carcinoma (Eynden, 2005), basal-like (Savage *et al*, 2007, 2008) and triple-negative breast carcinoma (Shack *et al*, 2003; Tan *et al*, 2007; Savage *et al*, 2007, 2008). Aside from breast, the association between caveolin expression and poor patient outcome was noticed in other tissue tumours (Savage *et al*, 2007, 2008), including prostate (Karam *et al*, 2007), lung ((Ho *et al*, 2008), and the central nervous system (Barresi *et al*, 2006).

The mechanism underlying the expression of CAV1 and CAV2 in breast cancer and specifically the basal-like phenotype is yet to be determined. In a small proportion of basal-like breast cancers, this seems to be driven by gene amplification (Savage *et al*, 2007, 2008). Moreover, hypomethylation of *CAV1* and *CAV2* promoters may be a possible cause (Engelman *et al*, 1999). However, the mechanism for CAV1 and CAV2 expression in basal-like cancers may stem from maintenance of a basal/myoepithelial phenotype or might be part of a transcriptomic programme of myoepithelial/basal-like differentiation, as these proteins are preferentially expressed in basal/myoepithelial cells of normal breast.

In conclusion, our findings and those of other recently reported studies show that CAV1 and CAV2 have oncogenic properties and are associated with breast carcinomas of basal-like and triple negative phenotypes. On univariate analysis, expression of caveolins was significantly associated with high histological grade, poor Nottingham Prognostic Index and with a more aggressive clinical behaviour (i.e. CAV2 expression was associated with shorter DFS in the whole cohort and CAV1 was associated with short BCSS in the group of low grade tumours). However, on multivariate analysis, both proteins were shown not to be independent prognostic factors for DFS and BCSS. Further studies are warranted to identify whether CAV1 and CAV2 play a role in the biology of basal-like and triple-negative tumours, or if they are mere surrogate markers for basal differentiation.

## Figures and Tables

**Table 1:** The frequency of CAV1 and CAV2 expressions in invasive breast carcinoma

	**Number of positive cases**	**Percentage**	**Total number**
CAV1	62	13.4	461
CAV2	24	5.9	410
CAV1 and CAV2	8	2.5	310

**Table 2:** Correlation between expression of CAV1 and CAV2 in tumor cells and clinicopathological and immunohistochemical markers in invasive breast cancer

	**CAV1**				**CAV2**			
**Parameter**	**Number of samples**	**Negative**	**Positive**	***P*-value**	**Number of samples**	**Negative**	**Positive**	***P*-value**
Grade	449			**0.026**	400			**0.005** ^*^
1		78	6			72	1	
2		126	14			111	2	
3		186	39			193	21	
								
Size	449			0.100	400			**<0.001** ^**^
⩽2 cm		236	36			238	6	
>2 cm		154	23			138	18	
								
LN	447				399			0.259
Negative		250	41	0.558		242	18	
Positive		138	18			134	5	
								
NPI	447			0.112	399			**0.017** ^*^
Good		125	12			115	1	
Moderate		202	39			203	18	
Poor		61	8			58	4	
								
Vascular invasion	442			1.000	319			0.369
No		270	42			250	14	
Yes		113	17			117	10	
								
ER	431			**<0.001** ^**^	384			**<0.001** ^**^
Negative		113	37			126	18	
Positive		264	17			237	3	
								
PR	429				386			**<0.001** ^**^
Negative		117	38	**0.004** ^**^		171	20	
Positive		197	17			193	2	
								
AR	408			0.140	364			**0.039** ^**^
Negative		144	28			143	14	
Positive		210	26			200	7	
								
Ck7/8	445			**0.003** ^**^	395			**<0.001** ^**^
Negative		142	34			128	17	
Positive		244	25			243	7	
								
Ck18	402			**0.026** ^**^	363			**0.022** ^**^
Negative		97	24			92	11	
Positive		250	31			250	10	
								
Ck19	442			**0.001** ^**^	391			0.161
Negative		75	23			65	7	
Positive		310	34			303	16	
								
Ck5/6	444			**0.004** ^**^	396			**<0.001** ^**^
Negative		296	34			298	8	
Positive		89	25			74	16	
								
Ck14	434			**0.038** ^**^	383			**0.001** ^**^
Negative		302	39			293	12	
Positive		74	19			66	12	
								
P-Cadherin	354			**0.007** ^**^	319			0.230
Negative		119	8			103	4	
Positive		190	37			195	17	
								
FGFR1	289			0.491	256			1.000
Negative		235	28			221	13	
Positive		25	1			21	1	
								
P63	445			**<0.001** ^**^	397			**<0.001** ^**^
Negative		378	50			365	17	
Positive		9	8			8	7	
								
E- cadherin	429			0.060	387			**0.034**
Negative		241	44			221	18	
Positive		130	14			144	4	
								
EGFR1	314	215	37	0.084	279			0.74
Negative		54	8			212	13	
Positive						52	2	
								
Nielsen groups	367			**<0.001** ^*^	320			**<0.001** ^*^
Her2		46	8			44	3	
Luminal		243	17			221	2	
Basal-like		36	17			40	10	
								
TN	430			**<0.001** ^**^	386			**<0.001** ^**^
Non TN		314	33			298	9	
TN		60	23			66	13	

Significant *P*-values are in bold. ^*^χ^2^ test; ^**^, Fisher's exact test. Nielsen groups: HER2 (HER2-positive, ER any, Ck5/6 or EGFR any), basal-like (HER2-negative, ER-negative, Ck5/6 or EGFR positive), luminal (HER2-negative, ER-positive, Ck5/6 or EGFR any). LVI, lymph vascular invasion. TN: Triple-Negative phenotype.
